# Exercise May Increase Oxidative Stress in the Sciatic Nerve in Streptozotocin-Induced Diabetic Rats

**DOI:** 10.3390/medicina60030480

**Published:** 2024-03-14

**Authors:** Koji Nonaka, Junichi Akiyama, Satsuki Une

**Affiliations:** 1 Faculty of Health Sciences, Naragakuen University, Nara 631-8524, Nara, Japan; 2 Department of Physical Therapy, School of Health Care and Social Welfare, Kibi International University, Takahashi 716-8505, Okayama, Japan; akiyama@kiui.ac.jp; 3 Faculty of Education, Kagawa University, Takamatsu 760-8521, Kagawa, Japan; une.satsuki@kagawa-u.ac.jp

**Keywords:** diabetes mellitus, peripheral neuropathy, exercise, oxidative stress, antioxidant enzymes, neurotrophic factors

## Abstract

*Background and Objectives:* Diabetic peripheral neuropathy (DPN) affects approximately half of patients with diabetes mellitus (DM), contributing to falls and fractures. Oxidative stress, which is linked to DM-induced hyperglycemia, has been implicated in the onset of DPN. Although exercise is recommended for patients with DM, its effect on DPN remains unclear. Therefore, this study aimed to investigate the effect of exercise on DPN and the mechanisms involved. *Material and Methods*: Thirty male Wistar rats were divided into control, streptozotocin (STZ)-induced diabetic (DM), and STZ-induced diabetic/exercise (DM + Ex) groups. Diabetes was induced using STZ injection. Rats in the DM + Ex groups underwent six weeks of treadmill exercise. Sciatic nerve parameters, which included motor nerve conduction velocity (MNCV), antioxidant enzymes (catalase, glutathione peroxidase [GPx], and superoxide dismutase [SOD]), oxidative stress markers (malondialdehyde [MDA] and 4-hydroxy-2-nonenal [4HNE]), and neurotrophic factors (brain-derived neurotrophic factor [BDNF] and nerve growth factor [NGF]), were examined. *Results:* Exercise alleviated DM-induced decreases in MNCV in rats. Although exercise did not significantly affect antioxidant enzyme activity, 4HNE levels increased significantly, indicating increased oxidative stress. Additionally, exercise did not significantly affect DM-induced increases in NGF and BDNF levels in rats. *Conclusions:* Exercise may prevent DPN in rats with DM, possibly through nonantioxidant mechanisms.

## 1. Introduction

Diabetes mellitus (DM) is a condition characterized by sustained high blood sugar levels. This can happen when insulin secretion decreases, insulin sensitivity decreases, or both. The number of diabetes patients are steadily rising and are estimated to reach 642 million by the year 2040 [1]. Diabetes can lead to various complications, including neuropathy, kidney damage, and retinopathy, among others.

Peripheral neuropathy is the most common complication in DM [2], with approximately 50% of patients with DM suffering from diabetic peripheral neuropathy (DPN) [3]. DPN accelerates the loss of skeletal muscle mass and increases the risk of falls and fractures [4]. Therefore, it is important to delay the development and progression of DPN to prevent fractures from falls and reduce the risk of morbidity and mortality [5].

Oxidative stress is believed to be involved in DPN onset. Hyperglycemia activates lipid peroxidation and induces the overproduction of reactive oxygen species (ROS) in the murine sciatic nerve [6]. Oxidative stress has been shown to induce dysfunction of murine sciatic nerves [7]. For example, treatment with the antioxidant agent resveratrol attenuated DM-induced oxidative stress and ameliorated DPN in the sciatic nerves of rats [8]. Therefore, decreased oxidative stress may delay the development or onset of DPN.

Regular moderate-intensity aerobic exercise is recommended for patients with DM to prevent DPN [9], suggesting that aerobic exercise may have a positive effect on DPN [10]. However, the specific underlying mechanisms of exercise in DPN, especially on DM-induced oxidative stress, remain unclear. Exercise increases antioxidant enzymes in the heart [11,12], hippocampus [13], and skeletal muscle [14]. Additionally, exercise decreases oxidative stress markers in the heart [12], hippocampus [13], kidneys [15], and liver [16]. Based on these findings, it can be speculated that exercise increases antioxidant enzyme levels and decreases oxidative stress in several tissues in rats with DM. Therefore, we hypothesized that aerobic exercise increases antioxidant enzyme activity and decreases oxidative stress markers in the peripheral nerves of patients with DM.

To confirm this hypothesis, this study aimed to investigate the effect and potential mechanisms of exercise on DM-induced oxidative stress and DPN. Specifically, we examined motor nerve conduction velocity (MNCV), which is an index of DPN; the activities of the antioxidant enzymes catalase, glutathione peroxidase (GPx), and superoxide dismutase (SOD); and the expression of the oxidative stress markers malondialdehyde (MDA) and 4-hydroxy-2-nonenal (4HNE). Additionally, we measured the levels of the neurotrophic factors, brain-derived neurotrophic factor (BDNF) and nerve growth factor (NGF), which are believed to regulate the activity of antioxidant enzymes.

## 2. Materials and Methods

### 2.1. Animals

Thirty male Wistar rats (12 weeks old) were housed in a temperature-controlled room maintained at 22 °C under a 12 h light–dark cycle and were allowed free access to food and water. The rats were randomly divided into three groups with ten animals per group: control (Cont; *n* = 10), diabetic (DM; *n* = 10), and diabetes/exercise (DM + Ex; *n* = 10). Diabetes was induced via a single intraperitoneal injection of streptozotocin (STZ) (40 mg/kg body weight; Wako Pure Chemical Industries, Ltd., Osaka, Japan) dissolved in physiological saline, according to a previously described protocol with minor modifications [17]. In our previous study [17], STZ administration at 50 mg/kg caused severe symptoms; therefore, in this study, we decided to administer STZ at 40 mg/kg. Rats in the nondiabetic group were injected with equivalent amounts of blank vehicle. Before STZ injection and after 1, 3, and 5 weeks of STZ injection, blood was collected from the tail vein, and nonfasting blood glucose concentrations were measured using Glutest Ace R (Sanwa Kagaku Kenkyusyo, Co., Ltd., Nagoya, Japan). Rats with blood glucose concentrations >300 mg/dL were defined as diabetic. The body weight of the rats was measured before STZ injection and after 1, 2, 3, 4, 5, and 6 weeks of STZ injection.

This study was approved by the Institutional Animal Care and Use Committee of the Kibi International University (No. A18-05).

### 2.2. Exercise Protocol

Exercise was performed from the day after STZ administration. The exercise protocol was conducted as previously described [18,19]. Briefly, rats in the DM + Ex group were transferred to a motor-driven treadmill (Muromachi Kikai Co., Ltd., Tokyo, Japan) once a day, five days a week, for six weeks. The exercise intensity and duration were as follows: 10 m/min for 10 min in the first week, 10 m/min for 20 min in the second week, 14–15 m/min for 20 min in the third week, 14–15 m/min for 30 min in the fourth week, and 17–18 m/min for 30 min in the fifth and sixth weeks.

### 2.3. Motor Nerve Conduction Velocity (MNCV)

MNCV was measured via a noninvasive procedure using a Neuropack (MEB-2306; Nihon Koden Co., Tokyo, Japan), according to previously described procedures [20]. Briefly, rats were anesthetized with isoflurane and placed on a heating plate (temperature, 37 °C) to maintain body temperature. The sciatic nerve was first stimulated at the sciatic notch, followed by stimulation at the Achilles tendon. Stimulation was performed supramaximally using bipolar needle electrodes, and the evoked potentials were recorded from the interosseous muscle using a unipolar needle electrode. MNCV was calculated by dividing the distance between two stimulated points by the difference between the distal latency and proximal latency, which were obtained by stimulating the Achilles tendon and sciatic notch, respectively. MNCV was measured bilaterally, and the average value was used for the analysis. In rats in which the MNCV could only be measured on one side, the MNCV on the measured side was used for the analysis.

### 2.4. Sciatic Nerve Sampling, Homogenization, and Protein Content Determination

In brief, rats were anesthetized with an overdose of sodium pentobarbital (150 mg/kg) administered via intraperitoneal injection. The sciatic nerve was removed and stored at −80 °C until further analysis. The sciatic nerves were homogenized in ice-cold extraction buffer (20 mM Tris-HCl, pH 7.6, 25 mM KCl, and 1% Triton X-100) containing complete protease inhibitor cocktail (Roche Diagnostics, Tokyo, Japan). Homogenates were centrifuged at 12,000× *g* for 10 min at 4 °C, and aliquots of supernatants were used for subsequent analyses. Protein concentrations of the aliquots were measured using a Coomassie Protein Assay Kit (Thermo Fisher Scientific K.K., Yokohama, Japan). The absorbance was measured at 595 nm using a microplate reader (SH-1200Lab; Corona Electric Co., Inc., Ibaraki, Japan) to determine protein concentration.

### 2.5. Antioxidant Enzyme Activities

Catalase activity in each muscle was measured at 37 °C using a microplate reader, according to the method described by Li [21]. Catalase activity is expressed as U/mg of protein. GPX activity was measured using an NWLSS Glutathione Peroxidase Assay Kit (Northwest Life Science Specialties, LLC, Vancouver, WA, USA) according to the manufacturer’s instructions. GPX activity is expressed as mU/mg of protein. SOD activity was measured using an SOD Assay Kit (Dojindo Molecular Technologies, Inc., Rockville, MD, USA) according to the manufacturer’s instructions. SOD activity is expressed as U/mg of protein.

### 2.6. Western Blot Analysis

In brief, samples were lysed using EzApply (ATTO, Tokyo, Japan) under boiling for 5 min to extract the proteins. Proteins were separated via electrophoresis using 15% polyacrylamide gel (ATTO, Tokyo, Japan) and transferred to PVDF membranes (ATTO, Tokyo, Japan) using a semi-dry blotting method. Membranes were stained with Ponceau S staining solution (Beacle, Inc., Kyoto, Japan), scanned, destained, and blocked with EzBlock Chemi (ATTO) for 1 h at room temperature. Thereafter, the membranes were incubated at 37 °C for 1 h with the following primary antibodies: anti-NGF (1:10,000; bs-0067R; Bios, Woburn, MA, USA), anti-BDNF (1:10,000; E-AB-10500; Elabscience Biotechnology Inc., Houston, TX, USA), anti-MDA (1:10,000; sc-130087; Santa Cruz, CA, USA), and anti-4HNE (1:5000; MHN-020P; Japan Institute for the Control of Aging, Shizuoka, Japan). After three washes (10 min/wash) with EzWash (ATTO) containing 0.1% Tween 20 (TTBS), the membranes were incubated for 1 h at room temperature with the following horse radish peroxidase-conjugated secondary antibodies: anti-mouse IgG (1:25,000; 01803-44; Nacalai Tesque, Kyoto, Japan) for 4HNE, anti-rabbit IgG (1:25,000; 01827-44; Nacalai Tesque) for NGF and BDNF, and anti-goat IgG (1:20,000; ab97100; Abcam, Tokyo, Japan) for MDA. Finally, the membranes were washed three times (10 min/wash) with TTBS and reacted with ECL™ Prime Western Blot Detection Reagent (CE Healthcare UK Ltd., Buckinghamshire, England) for 5 min at room temperature, and the protein bands were obtained by using LumiCube (Liponics, Inc., Tokyo, Japan). The protein bands were analyzed using the JustTLC software ver. 4.0.3 (Sweday, Sondra Sandby, Sweden). Bands from the Ponceau-stained membranes were used as protein loading controls. The data were normalized to the control values.

### 2.7. Data Analysis

A rat that did not develop diabetes and rats that died during the experimental period were excluded; in total, analyses were performed on 10 rats in the Cont group, 9 rats in the DM group, and 8 rats in the DM + Ex group. All data are expressed as mean ± standard error (SE). Blood glucose and body weight data were compared among the three groups using two-way repeated measures analysis of variance (ANOVA), followed by Tukey’s post-hoc test to determine significant differences between the DM, Cont, and DM + Ex groups. Catalase, GPx, SOD, MDA, 4HNE, NGF, and BDNF data were compared among the three groups using one-way ANOVA, followed by Dunnett’s post-hoc test to determine significant differences between the groups. Statistical analyses were performed using Ekuseru-Toukei 2008 (Social Survey Recunk Information Co., Ltd., Tokyo, Japan) or SPSS ver. 25.0 (IBM Japan, Ltd., Tokyo, Japan). Statistical significance was set at *p* < 0.05. The effect sizes of catalase, GPx, SOD, MDA, 4HNE, NGF, and BDNF between the DM, Cont, and DM + Ex groups were calculated using Cohen’s d coefficient [22]. An effect size > 0.8 was considered large, whereas those of 0.5−0.8 and 0.2−0.5 were considered medium and small, respectively [22].

## 3. Results

### 3.1. Blood Glucose Concentration

**Figure 1:**
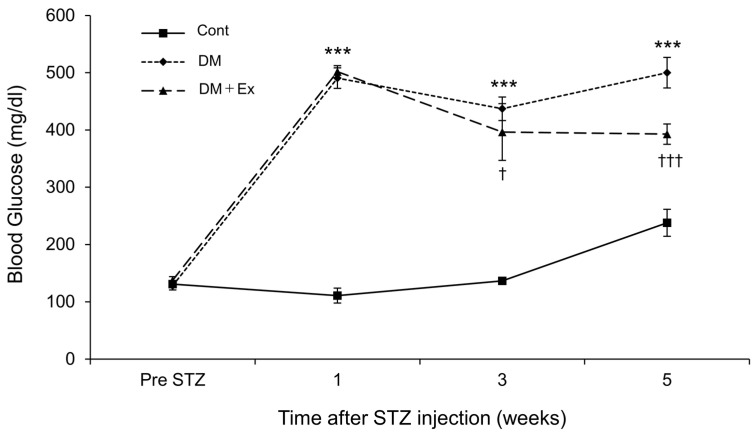
Changes in blood glucose concentration in rats in the control (Cont), diabetes (DM), and diabetes/exercise (DM ± Ex) groups. Values are presented as mean ± standard error. *** *p* < 0.001 vs. the Cont group, and † *p* < 0.05 and ††† *p* < 0.001 vs. the DM group at each time point.

The changes in blood glucose concentrations after exercise are shown in Figure 1. After STZ administration, blood glucose levels in the DM and DM + Ex groups were significantly higher than those in the Cont group. However, the DM + Ex group had significantly lower blood glucose levels than the DM group at three (*p* = 0.017) and five (*p* < 0.001) weeks after DM induction. Overall, these results suggest that treadmill exercise decreases blood glucose levels in rats with DM.

### 3.2. Body Weight

**Figure 2:**
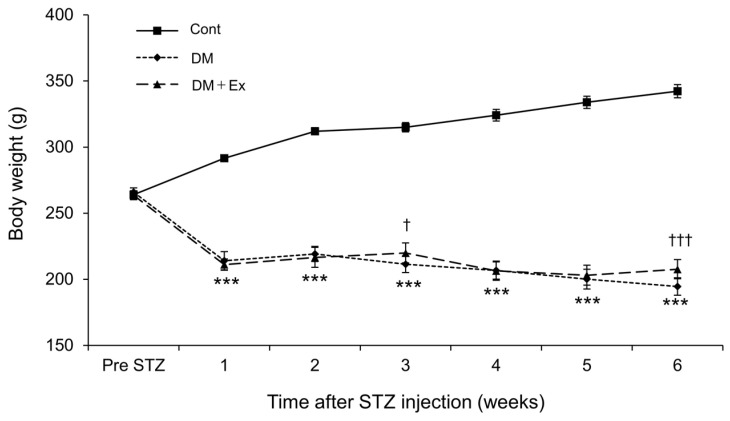
Changes in the body weight of rats in the control (Cont), diabetes (DM), and diabetes/exercise (DM ± Ex) groups. Data are presented as mean ± standard error. *** *p* < 0.001 vs. the Cont group, and † *p* < 0.05 and ††† *p* < 0.001 vs. the DM group at each time point.

Figure 2 shows the changes in body weight of the rats. Rats in the DM and DM + Ex groups had significantly lower body weights than those in the Cont group after STZ administration. However, rats in the DM + Ex group had significantly higher body weight than those in the DM group at three and six weeks after DM induction (*p* = 0.019 and *p* < 0.001, respectively). Collectively, these results suggest that treadmill exercise may alleviate DM-induced body weight loss.

### 3.3. MNVC

MNVC was measured to verify the degree of neuropathy and the effect of exercise on neuropathy in rats with DM (Table 1). MNVC was significantly lower in the DM group than in the control group, with a large effect size (d = 4.25). In contrast, MNCV was significantly higher (*p* = 0.047) in the DM + Ex group than in the DM group, and the effect size was large (d = 1.06). Overall, these results suggest that treadmill exercise may alleviate DM-induced DNP in rats.

### 3.4. MDA and 4HNE Contents

**Figure 3:**
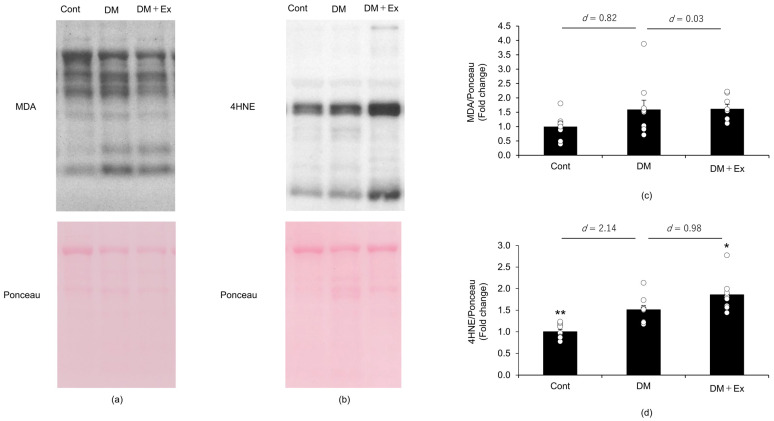
Malondialdehyde (MDA) and 4-hydroxy-2-nonenal (4HNE) levels in the sciatic nerves of the rats with diabetes, as indicated by Western blotting. MDA levels in the sciatic nerves of the rats in the control (Cont; *n* = 10), diabetes (DM; *n* = 9), and diabetes/exercise (DM ± Ex; *n* = 8) groups are shown in (**a**), and the mean ± standard error and the effect size d between the DM group and the Cont and DM ± Ex groups are shown in (**c**). 4HNE concentrations in the sciatic nerves of the rats in the three groups are shown in (**b**), and the mean ± standard error and the effect size d between the DM group and the Cont and DM ± Ex groups are shown in (**d**). * *p* < 0.05 and ** *p* < 0.01 vs. the DM group.

MDA and 4HNE levels were measured by using Western blotting to determine the effect of exercise on oxidative stress levels in the sciatic nerves of rats with DM, and the results are shown in Figure 3. Although there was no significant difference in MDA levels between the groups, the effect size between the control and DM groups was large (d = 0.82). However, the effect size for MDA between the DM and DM + Ex groups was small (d = 0.03). Collectively, these results suggest that MDA showed an increasing trend in the DM group compared with the control group, and that exercise did not affect DM-induced increases in MDA levels in the sciatic nerves of rats. Additionally, 4HNE levels were significantly higher (*p* = 0.002) in the DM group than in the Cont group, with a large effect size (d = 2.14). However, 4HNE levels were significantly higher (*p* = 0.047) in the DM + Ex group than in the DM group, with a large size effect (d = 0.98). Overall, these results indicate that treadmill exercise enhances DM-induced increases in 4HNE levels in the sciatic nerve of rats.

### 3.5. Antioxidant Enzyme Activities

Catalase, GPx, and SOD activities were measured to determine the effects of exercise on antioxidant enzymes in the sciatic nerves of rats with DM (Table 2). Although there was no significant difference in catalase activity between the groups, a large effect size (d = 1.00) was observed between the control and DM groups. In contrast, the effect size for catalase activity was small (d = 0.35) between the DM and DM + Ex groups. These results suggest that there was a decreasing trend in catalase activity in the sciatic nerve of rats with DM compared with those in the control group, and that exercise had a minimal effect on catalase activity. Similarly, although there was no significant difference in GPx activity between the groups, a large effect size was observed between the Cont and DM groups (d = 0.76). In contrast, the effect size for GPx activity was small between the DM and DM + Ex groups (d = 0.17). Overall, these results suggest that there was a decreasing trend in GPx activity in the sciatic nerves of rats with DM and that exercise had a minimal effect on GPx activity. Furthermore, SOD activity was significantly higher (*p* = 0.019) in the sciatic nerve of rats in the DM group than of those in the control group, and the effect size was large (d = 1.12). However, there was no significant difference (*p* = 0.656) in SOD activity between the DM and DM + Ex groups, and the effect size was small (d = 0.36). Collectively, these results suggest that exercise does not affect DM-induced increases in SOD activity in the sciatic nerve of rats.

### 3.6. NGF and BDNF Proteins

**Figure 4:**
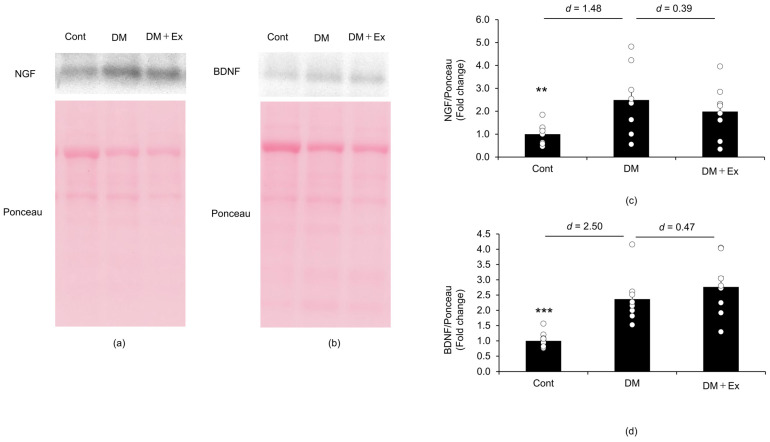
Nerve growth factor (NGF) and brain-derived neurotrophic factor (BDNF) levels in the sciatic nerves of rats with diabetes, as indicated by Western blotting. NGF protein expression in the sciatic nerves of rats in the control (Cont; *n* = 10), diabetes (DM; *n* = 9), and diabetes/exercise (DM ± Ex; *n* = 8) groups is shown in (**a**), and the mean ± standard error and the effect size d between the DM group and the Cont and DM ± Ex groups are shown in (**c**). BDNF protein expression in the sciatic nerves of rats in the three groups is shown in (**b**), and the mean ± standard error and the effect size d between the DM group and the Cont and DM ± Ex groups are shown in (**d**). ** *p* < 0.01 and *** *p* < 0.001 vs. the DM group.

Western blotting was performed to investigate the effect of exercise on NGF and BDNF levels in the sciatic nerves of rats with DM (Figure 4). The NGF protein level was significantly higher (*p* < 0.01) in the sciatic nerve of rats in the DM group (2.49 ± 0.46) than of those in the control group (1.00 ± 0.14), with a large effect size (d = 1.48). In contrast, there was no significant difference (*p* = 0.521) in NGF levels between the DM (2.49 ± 0.46) and DM + Ex (1.99 ± 0.41) groups, and the effect size was small (d = 0.39). These results suggest that exercise does not affect DM-induced increases in NGF levels in the sciatic nerve of rats. Additionally, the BDNF level was significantly higher (*p* < 0.001) in the sciatic nerve of rats in the DM group (2.37 ± 0.25) than of those in the control group (1.00 ± 0.08), with a large effect size (d = 2.50). However, there was no significant difference (*p* = 0.401) in NGF levels in the sciatic nerve between the DM (2.37 ± 0.25) and DM + Ex (2.77 ± 0.34) groups, and the effect size was small (d = 0.47). Collectively, these results suggest that exercise does not affect DM-induced increases in BDNF levels in the sciatic nerve of rats.

## 4. Discussion

In this study, we investigated the effects of exercise on DPN and oxidative stress in the peripheral nerves of rats with DM. Exercise suppressed DM-induced decreases in MNCV in the sciatic–tibial nerves of rats with DM. In contrast, exercise did not increase antioxidant enzyme activity in the sciatic nerves of rats with DM but increased the levels of one of the markers of oxidative stress. These findings suggest that exercise may prevent DPN but may increase oxidative stress in the peripheral nerves.

DM induced an increase in NGF and BDNF levels in the sciatic nerve of diabetic rats. DM has been reported to reduce the protein expression of NGF [23] and BDNF [24] in the sciatic nerve of rats; these findings did not correspond to our results. However, an increase in NGF gene expression was observed in the sciatic nerves of rats with DM [25]. Moreover, high glucose levels caused an increase in the mRNA and protein levels of NGF and BDNF in Schwann cells in vitro [26]. Although the reason for the discrepancies in NGF and BDNF levels between the present study and previous studies is unclear [23,24], the conditions of the model animals may have been different.

Treadmill exercise increased NGF and BDNF mRNA in the motor and sensory roots of sciatic nerve spinal segments of diabetic rats [19]. These findings suggested that exercise increases NGF and BDNF in rats with DM, but this did not correspond to our results. As stated above, while previous reports have suggested decreases in the levels of NGF [23] and BDNF [24] in the sciatic nerve of rats with DM, these proteins were decreased in the sciatic nerve of the rats with DM in the present study. Here, rats with DM might have already possessed high NGF and BDNF levels in the sciatic nerve. This may be the reason why exercise could not increase NGF and BDNF levels in the sciatic nerve of rats with DM.

DM has been shown to cause a decrease in the activities of catalase, GPx, and SOD in the sciatic nerves of rats [23]. Contrary to previous findings [23], DM did not significantly affect catalase and GPx activities in the sciatic nerves of the rats in this study; however, there was an increase in SOD activity. Al-Rejaie et al. [23] reported a decrease not only in antioxidant enzyme activities but also in the level of the NGF protein. NGF regulates and maintains catalase, GPx, and SOD activities in the brains of old and young rats [27]. Mattoson et al. [28] reported that NGF increased catalase activity, and BDNF increased GPx and SOD activities in the hippocampus of rats. Additionally, NGF has increased catalase activity in PC12 cells in vitro [29]. Based on these results, it could be concluded that NGF and BDNF regulate antioxidant enzyme activity. In the present study, there was an increase in NGF and BDNF levels in the sciatic nerves of rats with DM. Moreover, it is possible that the DM-induced increases in NGF and BDNF levels did not affect the activity of antioxidative enzymes in the sciatic nerve of rats with DM.

In the present study, exercise did not alter catalase, GPx, or SOD activity in the sciatic nerves of rats with DM. A previous study showed that exercise increased catalase and SOD activities in the sciatic nerve of rats with DM [30]. Additionally, exercise has increased SOD mRNA in the sciatic nerve of rats with DM [31]. These findings suggest that exercise increases antioxidant enzymes, in contrast to the results of this study. Ghoweba et al. [31] reported that exercise increased not only SOD mRNA but also NGF levels in the sciatic nerve of rats with DM [31]. Here, exercise did not enhance NGF and BDNF levels in the sciatic nerves of rats with DM, which may be the reason why exercise failed to increase antioxidant enzymes.

Compared with the rats in the control group, there was an increase in the levels of the oxidative stress markers MDA and 4HNE in the sciatic nerves of rats with DM, confirming that DM increased oxidative stress in the sciatic nerve. However, there was no significant difference in the MDA levels between the DM and DM + Ex groups. Similarly, a previous study reported an increase in MDA levels in the sciatic nerve due to DM [32]. Contrary to our findings, Chis et al. [30] reported that swimming exercises for five weeks decreased MDA levels in the sciatic nerves of rats with DM. Additionally, Chen et al. [33] reported that treadmill exercise suppressed DM-induced increases in MDA levels in the sciatic nerve of rats 14 days after DM induction; however, exercise had no effect on MDA levels 28 days after DM induction. These findings suggest that the effect of exercise on MDA production in the sciatic nerve may vary depending on the type of exercise and the duration of DM. 4NHE levels were significantly higher in the DM + Ex group than in the DM group, indicating that exercise may increase oxidative stress in the sciatic nerve of rats with DM. Given that there was no significant difference in antioxidant enzyme activity between the DM and DM + Ex groups, it could be inferred that the increase in oxidative stress was because the antioxidant enzymes were unable to sufficiently eliminate ROS generated in the sciatic nerve during exercise. Urinary 8-hydroxydeoxyguanosine, an indicator of oxidative stress, increases after exercise in patients with severe chronic obstructive pulmonary disease [34], suggesting that exercise increases oxidative stress and may be harmful to patients with diseases that predispose them to oxidative stress. Given that patients with DM are prone to oxidative stress, exercise may further exacerbate oxidative stress. Therefore, exercise intensity should be regulated, and antioxidant supplementation may be considered in patients with DM to prevent oxidative stress.

In the present study, exercise attenuated DM-induced decreases in MNCV in rats with DM, suggesting that exercise may be effective in preventing neuropathy. Similarly, a previous study [35] showed that treadmill exercise for eight weeks attenuated DM-induced decreases in the MNCV of the sciatic–tibial nerve in rats with STZ-induced diabetes. Ghoweba et al. [31] also reported that the nerve conduction velocity (NCV) of the sciatic nerve was higher in rats with DM subjected to swimming exercises for four weeks compared to those with DM and without exercise. Additionally, Gholami et al. [36] reported that the NCV of the peroneal and tibial nerves improved after 12 weeks of aerobic exercise in diabetic patients. These results suggest that exercise may prevent or improve the decrease in nerve conduction velocity caused by DM. In contrast, Lee et al. [37] reported that swimming exercises immediately after DM induction did not significantly attenuate the decrease in caudal motor and sensory NCV in rats. Gholami et al. [36] reported that aerobic exercise did not improve tibial NCV in diabetic patients. Furthermore, diabetic patients who underwent aerobic exercise and unilateral lower limb resistance exercise for 10 weeks reported improvements in peroneal and tibial NCV [38]. However, while the NCV of the tibial nerve in diabetic patients improved with exercise, there was no interaction effect observed in the NCV of the tibial nerve between diabetic patients who exercised and those who did not [38]. These reports suggest that the prevention of or improvement in NCV decline that is induced by DM may vary depending on the type of nerve. In this study, the MNCV of sciatic–tibial nerves in rats with DM improved with exercise, but different results may be obtained for other nerves. Notably, NCV is related to myelin, and low-intensity treadmill exercise reduces myelin breakdown [39]. Therefore, it could be speculated that exercise attenuated DM-induced decreases in MNCV in rats in the present study by reducing myelin breakdown. Land-based exercises such as treadmill exercises can induce Schwan cell apoptosis [40]. Moreover, hyperglycemia-induced increases in oxidative stress cause Schwann cell apoptosis, which can be reduced by suppressing ROS production using the antioxidant melatonin [41]. Overall, these findings suggest that exercise may increase 4HNE levels in the sciatic nerves of rats with DM, which increases oxidative stress. Therefore, we cannot exclude the possibility that exercise may increase oxidative stress and Schwann cell apoptosis in DM. Exercise has been reported to increase NT-3, a neurotrophin, in muscles and is passively correlated with caudal NCV [42]. This suggests that exercise-induced increases in muscle NT-3 levels have neuroprotective effects. Therefore, exercise may have positive effects by increasing NT-3 levels in the muscles of DM rats or negative effects by increasing Schwann cell apoptosis due to increased oxidative stress. The effect of exercise on neuropathy in DM is believed to be determined by the balance between the positive and negative effects. Exercise reportedly attenuates the decrease in myelin sheet thickness in DM [43]. In this study, the positive effects may have outweighed the negative effects, resulting in a decrease in myelin organization and the attenuation of DM-induced decreases in MNCV.

Despite the promising findings, this study has some limitations. For example, exercise intensity may have been high, which increased oxidative stress markers in the sciatic nerves of rats with DM. Therefore, exercise intensity should be lower in patients with DM who are prone to oxidative stress, indicating the need for further studies on optimal exercise intensity. Additionally, although exercise prevented DM-induced decreases in MNCV in rats, it may increase oxidative stress. Therefore, the use of antioxidants combined with low-intensity exercises may be effective in preventing peripheral neuropathy. However, further studies are necessary to investigate the effects of combined antioxidant supplementation and exercise routine on peripheral neuropathy. Moreover, the results of this study suggest that the preventive effect of exercise on peripheral neuropathy may not be due to a decrease in oxidative stress, as there was no significant difference in the activities of antioxidant enzymes between the groups. Therefore, it is necessary to investigate other potential mechanisms through which exercise may prevent DM-induced peripheral neuropathy.

## 5. Conclusions

Based on the MNCV values, it could be concluded that exercise may delay the onset of DM-associated neuropathy or alleviate symptoms. However, exercise may have both positive and negative effects in rats with DM, as there was an increase in oxidative stress, without a corresponding increase in the activities of antioxidant enzymes.

## Figures and Tables

**Table 1:** Montor nerve conduction velocity (MNVC) of the sciatic–tibial nerve.

	Cont (*n* = 10)	DM (*n* = 9)	DM + Ex (*n* = 8)	One-Way ANOVA	Effect Size d Value	
				*p*-Value	Con vs. DM	DM vs. DM + Ex
MNVC (m/s)	45.1 ± 0.9	32.2 ± 1.1 ***	36.0 ± 1.4 ^†^	<0.001	4.25	1.06

Values are expressed as mean ± standard error. Cont, control; DM, diabetes; DM + Ex, diabetes/exercise. *** *p* < 0.001 vs. the Cont group; ^†^ *p* < 0.05 vs. the DM group.

**Table 2:** Catalase, glutathione peroxidase (GPx), and superoxide dismutase (SOD) activities in the sciatic nerve of rats.

	Cont (*n* = 10)	DM (*n* = 9)	DM + Ex (*n* = 8)	One-Way ANOVA	Effect Size d Value	
				*p*-Value	Con vs. DM	DM vs. DM + Ex
Catalase (U/mg of protein)	10.70 ± 0.93	8.20 ± 0.63	8.93 ± 0.82	0.092	1.00	0.35
GPx (mU/mg of protein)	0.66 ± 0.07	0.86 ± 0.10	0.81 ± 0.09	0.235	0.76	0.17
SOD (U/mg of protein)	1.01 ± 0.14	1.64 ± 0.22 *	1.46 ± 0.09	0.029	1.12	0.36

Values are expressed as mean ± standard error. Cont, control; DM, diabetes mellitus; DM + Ex, diabetes/exercise. * *p* < 0.05 vs. the Cont group.

## Data Availability

The data are available upon specific and reasonable request through direct contact with the corresponding authors.
